# Supplementary material for: Probable Pain on the Pain Assessment in Impaired Cognition (PAIC15) Instrument: Assessing Sensitivity and Specificity of Cut-Offs against Three Standards
Source: Brain Sci. 2021 Jun 29;11(7):869. doi: 10.3390/brainsci11070869 (PMC8301856; doi:10.3390/brainsci11070869)
Supplement: Supplementary file 1 [file brainsci-11-00869-s001.zip › brainsci-1246172-supplementary.pdf]

SUPPLEMENTAL FILES

Figures S1a-f. Six ROC figures (two assessments, three standards)

Conform Table 5 in the article, green circles indicate highest sensitivity plus specificity (coordinate of the ROC-curve with the most upper left position). Blue circles indicate sensitivity and specificity most balanced (values closest) for this cut-off.

(S1a)

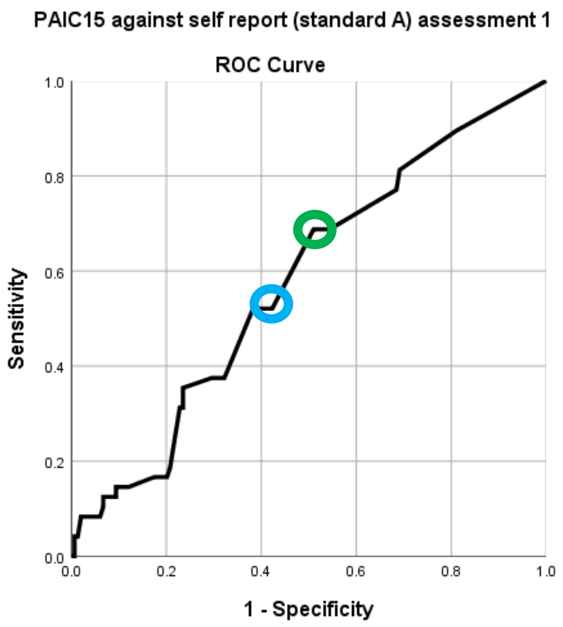

(S1b)

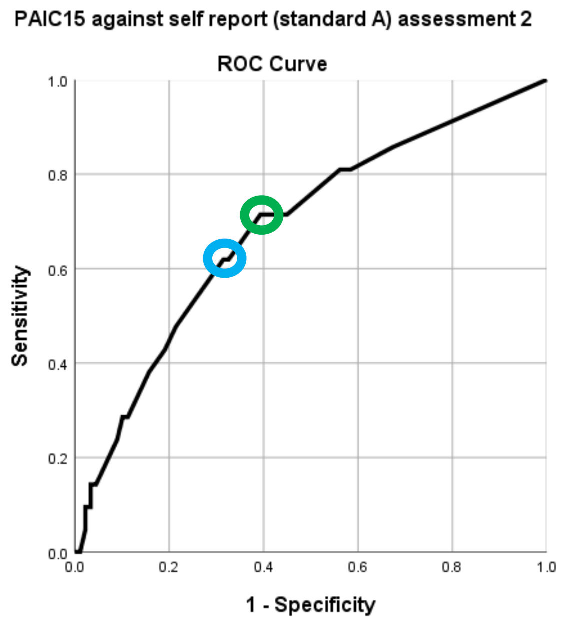

(S1c) PAIC15 against PAINAD (standard B) assessment 1

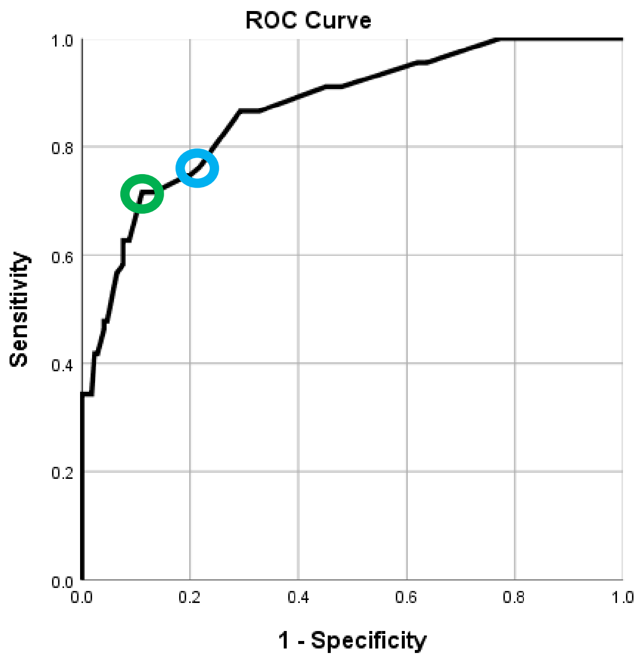

(S1d) PAIC15 against PAINAD (standard B) assessment 2

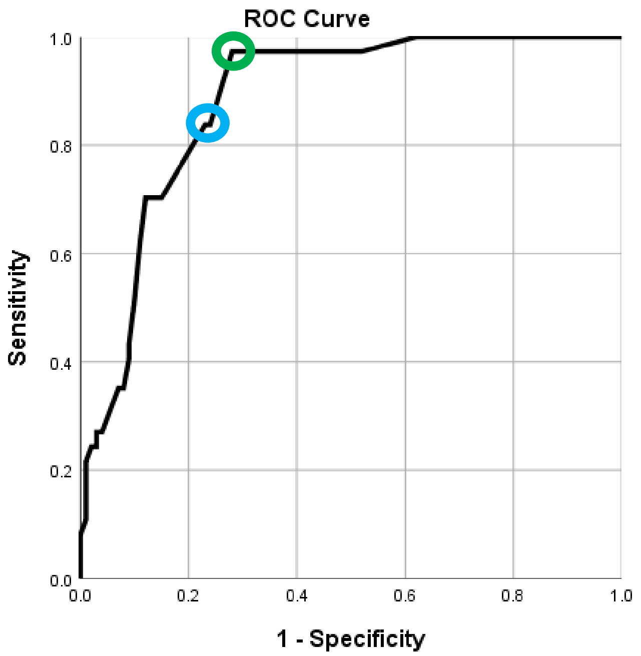

(S1e) PAIC15 against observer's overall estimate (standard C) assessment 1

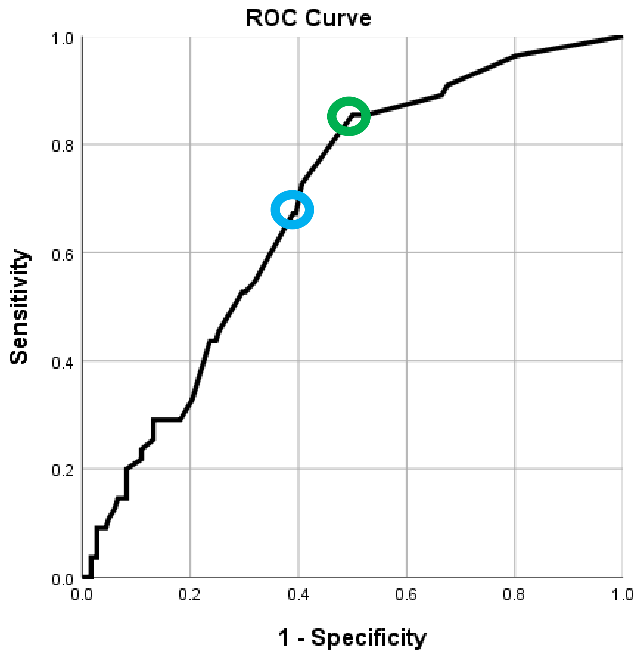

(S1f) PAIC15 against observer's overall estimate (standard C) assessment 2

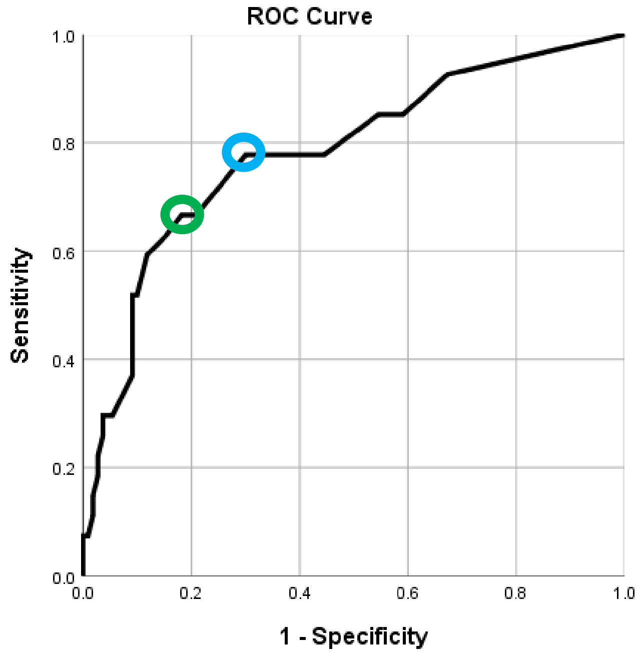

Box S1. Self-report and observer's estimate instruments used
Translated by professional translator or validated translation taken from MDS 3.0 (verbal pain descriptors)*

Self-report

Is the resident in pain at this moment? The resident's answer is:
☐ yes
☐ no
☐ the resident does not respond to this question

How *bad* is the pain at this moment? The resident's answer is:
☐ not applicable, the resident indicates not experiencing any pain
☐ a score of …… (between 0 and 10)
☐ the resident does not respond to this question

0	1	2	3	4	5	6	7	8	9	10
No pain	Mild pain				Moderate to severe pain					Very severe / horrible pain

*CMS Centers for Medicare & Medicaid Services. MDS 3.0 RAI Manual, 2019. https://www.cms.gov/Medicare/Quality-Initiatives-Patient-Assessment-Instruments/NursingHomeQualityInits/MDS30RAIManual (Accessed 2 April 2021)

Observer's estimate
Is the resident in pain at this moment? My assessment, *following* the observations and the question put to the resident, is:
☐ yes
☐ no

How *bad* is the pain at this moment? My assessment, *following* the observations and the question put to the resident, is:
a score of …… (between 0 and 10)

Table S1. PAIC15 items and item scores

PAIC15 item	Description	Item scores (theoretical item range 0-3)			
		Assessment 1 (n=238)		Assessment 2 (n=137)	
		Mean (SD)	N not scorable, missing otherwise	Mean (SD)	N not scorable, missing otherwise
Facial expression					
Frowning	lowering and drawing brows together	0.53 (0.78)	1, 0	0.40 (0.69)	0, 0
Narrowing eyes	narrowed eyes with tension around the eyes	0.34 (0.65)	0, 0	0.30 (0.64)	1, 0
Raising upper lip	upper lip raised, nose may be wrinkled	0.16 (0.47)	1, 0	0.10 (0.35)	0, 0
Opening mouth	the lips are parted, jaw is dropped	0.46 (0.83)	0, 0	0.37 (0.80)	0, 0
Looking tense	facial display of strain or worry	0.63 (0.86)	0, 0	0.61 (0.94)	0, 0
Body movements					
Freezing	stiffening, avoiding movement, holding breath	0.16 (0.51)	0, 0	0.19 (0.54)	0, 0
Guarding	protecting affected area, holding body part, avoiding touch, moving away	0.10 (0.42)	1, 0	0.14 (0.52)	2, 0
Resisting care	resisting being moved or resisting care, being uncooperative	0.21 (0.62)	41, 0	0.18 (0.60)	25, 0
Rubbing	tugging or massaging affected area	0.17 (0.50)	2, 0	0.16 (0.47)	2, 0
Restlessness	fidgeting, wringing hands, rocking back and forth	0.74 (0.99)	0, 0	0.57 (0.92)	0, 0
Vocalization					
Using pain-related words	using pain words, like “ouch”, “ow”, or “that hurts”	0.14 (0.48)	0, 0	0.15 (0.49)	0, 0
Shouting	using a loud voice to express words	0.14 (0.50)	0, 0	0.07 (0.38)	0, 0
Groaning	making a deep, inarticulate sound	0.14 (0.46)	0, 21*	0.12 (0.48)	0, 0
Mumbling	uttering words and/or sounds indistinctly	0.42 (0.77)	0, 0	0.28 (0.62)	0, 0
Complaining	expressing being unhappy, sick, uncomfortable, and/or in pain	0.25 (0.68)	0, 0	0.32 (0.70)	1, 0
**Total score**	Total score imputed with patient mean if maximum of 5 items missing	4.66 (5.13)	0	4.02 (5.22)	0

*In the first cohort, the item was missing in the data entry program and when available, we could abstract from the print version which was complete

Table S2. Sensitivity and specificity of PAIC15 cut-offs against the three standards

Assessment 1									Assessment 2								
PAIC15 score[1]	Self-report[2]		PAIC15 score[1]	PAINAD cut-off		PAIC15 score[1]	Observer's overall estimate		PAIC15 score[1]	Self-report[2]		PAIC15 score[1]	PAINAD cut-off		PAIC15 score[1]	Observer's overall estimate	
	Sensitivity	Specificity		Sensitivity	Specificity		Sensitivity	Specificity		Sensitivity	Specificity		Sensitivity	Specificity		Sensitivity	Specificity
0.5	0.896	0.188	0.50	1.000	0.228	0.5	0.964	0.198	0.5	0.857	0.326	0.5	1.000	0.380	0.5	0.926	0.327
1.0	0.813	0.309	1.0	0.955	0.363	1.0	0.909	0.324	1.0	0.810	0.416	1.0	0.973	0.480	1.0	0.852	0.409
1.5	0.771	0.315	1.5	0.955	0.380	1.5	0.891	0.335	1.5	0.810	0.438	1.1	0.973	0.520	1.1	0.852	0.445
2.1	0.688	0.456	2.1	0.910	0.520	2.1	0.855	0.473	2.1	0.714	0.551	1.6	0.973	0.530	1.6	0.852	0.455
2.2	0.688	0.470	2.2	0.910	0.532	2.2	0.855	0.484	2.6	0.714	0.607	2.1	0.973	0.660	2.1	0.778	0.555
2.7	0.688	0.490	2.7	0.910	0.550	2.7	0.855	0.500	3.1	0.619	0.674	2.6	0.973	0.720	2.6	0.778	0.609
3.1	0.521	0.577	3.1	0.866	0.673	3.1	0.727	0.593	3.6	0.619	0.685	3.1	0.838	0.760	3.1	0.778	0.691
3.3	0.521	0.611	3.3	0.866	0.702	3.3	0.673	0.604	4.1	0.476	0.787	3.6	0.838	0.770	3.6	0.778	0.700
3.7	0.521	0.617	3.7	0.866	0.708	3.7	0.673	0.610	4.6	0.429	0.809	4.1	0.703	0.850	4.1	0.667	0.791
4.1	0.375	0.678	4.1	0.761	0.784	4.1	0.545	0.681	5.5	0.381	0.843	4.6	0.703	0.880	4.6	0.667	0.818
4.5	0.375	0.698	4.5	0.746	0.801	4.5	0.527	0.698	6.5	0.333	0.865	5.5	0.622	0.890	5.5	0.630	0.845
4.8	0.375	0.705	4.8	0.746	0.807	4.8	0.527	0.703	7.3	0.286	0.888	6.5	0.514	0.900	6.5	0.593	0.882
5.2	0.354	0.765	5.2	0.716	0.865	5.2	0.455	0.747	7.8	0.286	0.899	7.3	0.432	0.910	7.3	0.519	0.900
5.6	0.313	0.765	5.6	0.716	0.877	5.6	0.436	0.753	8.5	0.238	0.910	7.8	0.405	0.910	7.8	0.519	0.909
5.9	0.313	0.772	5.9	0.716	0.889	5.9	0.436	0.764	9.3	0.143	0.955	8.3	0.351	0.920	8.3	0.407	0.909
6.2	0.188	0.792	6.2	0.627	0.912	6.2	0.345	0.791	10.8	0.143	0.966	8.8	0.351	0.930	8.8	0.370	0.909
6.7	0.167	0.799	6.7	0.627	0.924	6.7	0.327	0.797				9.3	0.270	0.960	9.3	0.296	0.945
7.3	0.167	0.819	7.3	0.582	0.924	7.3	0.309	0.808				10.3	0.270	0.970	10.3	0.296	0.955
7.8	0.167	0.826	7.8	0.567	0.936	7.8	0.291	0.819									
8.0	0.146	0.879	8.0	0.478	0.953	8.0	0.291	0.868									
8.5	0.146	0.886	8.3	0.478	0.959	8.3	0.273	0.868									
9.1	0.146	0.906	8.8	0.463	0.959	8.8	0.255	0.868									
9.6	0.125	0.906	9.1	0.418	0.971	9.1	0.236	0.890									
10.4	0.125	0.933	9.6	0.418	0.977	9.6	0.218	0.890									
.			10.2	0.343	0.982	10.2	0.200	0.918									

[1]Imputed PAIC15 values shown up to 10 and the values represent the coordinates of the ROC curves in this Supplemental file. [2]Pain and intensity 1 and up versus other for those who reported. In green: highest sensitivity plus specificity (coordinate of the ROC-curve with the most upper left position). In blue: sensitivity and specificity most balanced (values closest) for this cut-off. In yellow: higher sensitivity than specificity while also closest to balance. Red font: sensitivity lower than specificity.
